# IVA: accurate *de novo* assembly of RNA virus genomes

**DOI:** 10.1093/bioinformatics/btv120

**Published:** 2015-02-28

**Authors:** Martin Hunt, Astrid Gall, Swee Hoe Ong, Jacqui Brener, Bridget Ferns, Philip Goulder, Eleni Nastouli, Jacqueline A. Keane, Paul Kellam, Thomas D. Otto

**Affiliations:** ^1^Wellcome Trust Sanger Institute, Wellcome Trust Genome Campus, Cambridge, UK,; ^2^Department of Paediatrics, University of Oxford, Oxford, UK,; ^3^Division of Infection and Immunity, Faculty of Medical Sciences, University College London, London, UK and; ^4^Department of Virology, University College London Hospital NHS Foundation Trust, London, UK

## Abstract

**Motivation:** An accurate genome assembly from short read sequencing data is critical for downstream analysis, for example allowing investigation of variants within a sequenced population. However, assembling sequencing data from virus samples, especially RNA viruses, into a genome sequence is challenging due to the combination of viral population diversity and extremely uneven read depth caused by amplification bias in the inevitable reverse transcription and polymerase chain reaction amplification process of current methods.

**Results:** We developed a new de novo assembler called IVA (Iterative Virus Assembler) designed specifically for read pairs sequenced at highly variable depth from RNA virus samples. We tested IVA on datasets from 140 sequenced samples from human immunodeficiency virus-1 or influenza-virus-infected people and demonstrated that IVA outperforms all other virus *de novo* assemblers.

**Availability and implementation:** The software runs under Linux, has the GPLv3 licence and is freely available from http://sanger-pathogens.github.io/iva

**Contact:** iva@sanger.ac.uk

**Supplementary information:** Supplementary data are available at *Bioinformatics* online.

## 1 Introduction

**Fig. 1.**
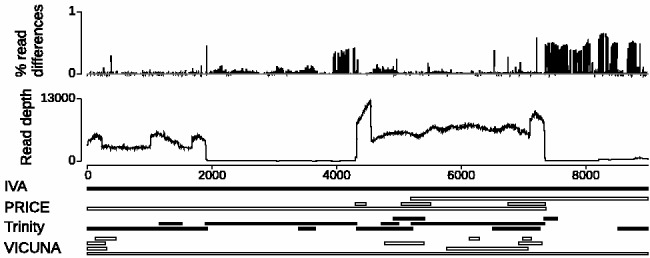
Example HIV-1 assemblies. Plots show the proportion of single base differences per mapped read compared to the IVA contig, the read depth and contigs from PRICE, Trinity and VICUNA aligned to the single IVA contig. The minimum read depth is 63

The main challenge of assembling sequence data from an RNA virus sample into a consensus sequence lies in the extremely variable read depth from current sequencing approaches combined with the extensive viral population diversity. An example is shown in Figure 1 where regions of the genome are represented with different read depths, caused by the separate reverse transcription polymerase chain reaction amplification of overlapping regions of the genome before library preparation. Further, there is a relatively high rate of single base differences in the reads throughout the genome. These properties of the data cause standard assembly algorithms to produce multiple contigs covering the same region and, more significantly, miss regions of the genome entirely (Yang *et al.*, 2012).

Despite the availability of at least 40 genome assemblers (http://en.wikipedia.org/wiki/Sequence_assembly), VICUNA (Yang *et al.*, 2012) and PRICE (Ruby *et al.*, 2013) are currently the only assemblers designed for virus data. VICUNA tackles the assembly problem by first clustering the reads that should belong to the same contig, using min hashes to infer similarity. Contigs are generated and then merged to form the final output. PRICE begins with seed sequences, which are iteratively extended by generating new sequence from local assemblies of reads at contig ends. In addition, the RNA-seq assembler Trinity (Grabherr *et al.*, 2011) has been used to assemble virus data because it can handle irregular read depth. Trinity constructs de Bruijn graphs from clusters of the reads, then resolves each cluster into transcripts by tracing reads and their mates through the graphs.

Our approach is similar to that of PRICE, except we extend contigs more conservatively using consensus kmers from the reads instead of using local assemblies. Also our new assembler, called IVA (Iterative Virus Assembler), is a completely *de novo* assembler, whereas PRICE must be provided with seed sequences to be extended into contigs.

## 2 Methods

A flowchart describing the assembly process is shown in Supplementary Figure S1 and full details are in the Supplementary Material. Before assembling, adapter sequences are removed from the reads using Trimmomatic (Bolger *et al.*, 2014), followed by the trimming of polymerase chain reaction primer sequences.

After trimming the reads, the most abundant kmer among the reads is found using kmc (Deorowicz *et al.*, 2013). This short seed kmer is iteratively extended into a contig using reads that have a perfect match to that kmer, treating the reads as unpaired. A list of all possible extension sequences is made (one sequence per overhanging read). IVA identifies the kmer of length *k* among prefixes of the possible extension sequences, for largest possible *k*, such that the kmer appears at least 10 times and is at least four times as abundant as the next most common kmer of length *k*. In this way, the seed is iteratively extended until its length reaches the insert size of the read pairs.

Contigs are extended in a similar manner to that of seed kmers. Instead of using perfect string matches, reads are mapped to the contigs with SMALT (http://www.sanger.ac.uk/resources/software/smalt/). During mapping, IVA also uses SAMtools (Li *et al.*, 2009). Reads mapped as part of a perfect pair (in the correct orientation and separated by the correct distance) and hang off a contig end are used to extend the contig. The sequence added to a contig end is constructed using the method described above for kmer extensions.

When no more contigs can be extended, they are cleaned as follows before generating a new seed. Contig ends are trimmed for quality, and overlapping contigs are merged based on sequence similarity found at their ends using nucmer (Kurtz *et al.*, 2004). Assembly stops either when a pre-defined maximum contig number is reached or no new seeds can be made.

## 3 Results

We evaluated IVA, PRICE, Trinity and VICUNA with different parameters on Illumina paired reads from 42 human immunodeficiency virus 1 (HIV-1) samples and 98 Influenza A and B virus samples. See the Supplementary Material for the full analysis. To compare the assemblies for each sample, we picked the closest reference from a pool of genomes using Kraken (Wood and Salzberg, 2014). For the accession numbers and complete evaluation procedure, see the Supplementary Material. We generated quality metrics using (i) nucmer to compare contigs with a reference genome, (ii) GAGE (Salzberg *et al.*, 2012) analysis code and (iii) RATT (Otto *et al.*, 2011) to transfer annotation from the reference to the assembly.

**Fig. 2.**
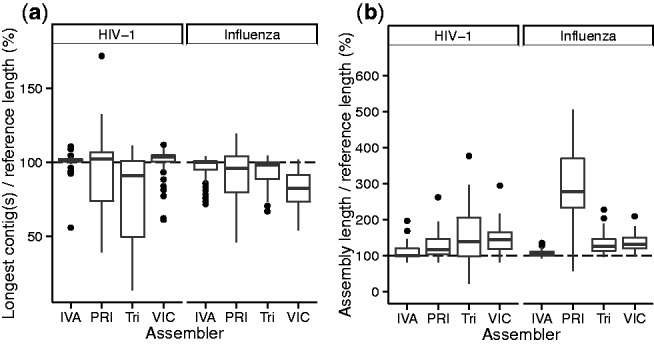
Comparison of assembly success. (**a**) For each segment of the reference, the longest matching contig was found. This plot shows the total length of these contigs for each assembly, as a percentage of the reference length. (**b**) Total assembly lengths, excluding contamination by only counting contigs that match the reference, as a percentage of the reference length

The ideal assembler output is defined as one contig for HIV-1, or exactly one contig for each Influenza virus genome segment, with the expected length compared to the closest reference and no duplication. IVA generated ideal assemblies for 57% of the HIV samples and 21% of the Influenza virus samples (Table 1 and Supplementary Tables S1 and S2), significantly more than the other assemblers. These low numbers are generally due to contigs of incorrect length (Fig. 2a) or duplications in the assemblies (Fig. 2b, Supplementary Figs S2 and S3, Table 1 and Supplementary Tables S1 and S2). IVA had the smallest variation in these results, especially for the Influenza virus samples (Fig. 2, Supplementary Figs S2 and S3, Table 1 and Supplementary Tables S1 and S2). The proportion of each reference genome assembled into contigs was 89.8-98.3% for HIV-1. The corresponding values for Influenza virus ranged from 89.8 (PRICE) to 98.8% (IVA). The mean per cent of HIV-1 annotation features transferred by RATT from IVA assemblies was 99.0% on both HIV-1 and Influenza virus samples. This was more than the other assemblers, except VICUNA with alternative settings that achieved 99.2% mean annotation transfer, at the expense of a duplication rate more than double that of IVA (Supplementary Table S1). There were few assembly errors—Trinity produced none, and IVA and VICUNA made one error each. The typical run time was under 10 h and none of the assemblers had excessive memory requirements (Supplementary Fig. S4). IVA was slightly slower on the HIV-1 samples but was comparable to PRICE and faster than VICUNA on the Influenza virus data.

**Table 1.** Summary of assembly QC results

	**HIV-1**				**Influenza**			
	IVA	PRI	Tri	VIC	IVA	PRICE	Tri	VIC
Ideal assemblies (%)^a^	57.1	11.9	14.3	2.4	21.4	0.0	1.0	0.0
Mean reference bases assembled (%)	97.9	97.2	89.8	98.3	98.8	89.8	97.6	94.3
Mean % annotation transferred	99.0	90.0	86.2	97.3	99.0	92.1	96.1	95.3
Total assembly errors^b^	1	4	0	1	0	6	0	0

^a^HIV-1: the entire genome must be assembled into a unique contig. Influenza: each segment must be assembled into a unique contig.

^b^An error is an inversion, relocation or translocation reported by GAGE. Numbers reported are the total across all assemblies. Supplementary Tables S1 and S2 expand on this table.

## 4 Discussion

Considering the number of ideal assemblies produced by the available tools, it can be seen that assembling RNA virus genomes is challenging. However, IVA was consistently better at producing single sequences representing the consensus sequence of each virus population, especially on the Influenza virus data. In contrast, the other tools tended to either produce multiple copies of parts of each genome or miss entire regions from their output. In summary, we developed IVA specifically to assemble short read sequencing data from RNA virus samples and have shown that it produces significantly higher quality assemblies than existing approaches.

## Supplementary Material

Supplementary Data
