# Supplementary material for: IVA: accurate de novo assembly of RNA virus genomes
Source: Bioinformatics. 2015 Feb 28;31(14):2374–6. doi: 10.1093/bioinformatics/btv120 (PMC4495290; doi:10.1093/bioinformatics/btv120)
Supplement: Supplementary Data [file supp_31_14_2374__index.html]

IVA: accurate de novo assembly of RNA virus genomes — IVA: accurate de novo assembly of RNA virus genomes — IVA: accurate de novo assembly of RNA virus genomes — Supplementary Data 

# IVA: accurate *de novo* assembly of RNA virus genomes

## Supplementary Data

files

**Files in this Data Supplement:**

- Supplementary Data - pdf file
- Supplementary Data - xls file
