# Supplementary material for: IVA: accurate de novo assembly of RNA virus genomes
Source: Bioinformatics. 2015 Feb 28;31(14):2374–6. doi: 10.1093/bioinformatics/btv120 (PMC4495290; doi:10.1093/bioinformatics/btv120)
Supplement: Supplementary Data [file supp_btv120_supplementary_material.pdf]

Supplementary Material of:

IVA: accurate *de novo* assembly of RNA virus genomes

Martin Hunt [1], Astrid Gall [1], Swee Hoe Ong [1], Jacqui Brener [2], Bridget Ferns [3], Philip Goulder [2], Eleni Nastouli [4], Jacqueline A Keane [1], Paul Kellam [1,3] and Thomas D Otto [1]

[1]Wellcome Trust Sanger Institute, Wellcome Trust Genome Campus, Cambridge, CB10 1SA, UK, [2]Department of Paediatrics, University of Oxford, Oxford, UK, [3]Division of Infection and Immunity, Faculty of Medical Sciences, University College London, London, UK, [4]Department of Virology, University College London Hospital NHS Foundation Trust, London, UK

Contents

1 Data and scripts **3**

2 IVA assembly methods **3**
- 2.1 Third-party software . . . 3
- 2.2 Illumina adapter and PCR primer trimming . . . 3
- 2.3 Seed generation . . . 4
- 2.4 Contig cleaning and merging . . . 4

3 Sample QC **4**
- 3.1 Description of test data . . . 4
- 3.2 Removing low quality samples . . . 5
- 3.3 Reference databases . . . 5

4 Benchmarking **6**
- 4.1 IVA . . . 6
- 4.2 VICUNA . . . 7
- 4.3 PRICE . . . 7
- 4.4 Trinity . . . 8

5 Assembly validation **8**

6 Run time and memory usage **9**

7 Example assemblies **9**

1 Data and scripts

The supplementary data and scripts developed specifically for this project can be found in the github repository https://github.com/sanger-pathogens/iva-publication. The Python 3 package Fastaq (https://github.com/sanger-pathogens/Fastaq) must be installed for the scripts in the iva-publication repository to work. The script `fastaq` is distributed with the Fastaq package, all other scripts are found in the `Scripts/` directory of the iva-publication repository.

The supplementary file `supplementary_tables.xls` contains supplementary Tables S3–S8. These tables are also available as tab-delimited plain text files in the github repository iva-publication.

2 IVA assembly methods

A flowchart describing the assembly algorithm used by IVA is shown in Figure S1.

2.1 Third-party software

The installation of IVA requires the user to install a set of third-party dependencies. IVA version 0.11.0 requires the following programs and versions:

- kmc version 2.1
- MUMmer version 3.23
- samtools version 0.1.19-44428cd
- SMALT version 0.7.6
- Trimmomatic version 0.32.

2.2 Illumina adapter and PCR primer trimming

IVA can optionally trim Illumina adapters and PCR primer sequences from the reads before assembling. Illumina adapters are removed from a pair of FASTQ files (`in.1.fq` and `in.2.fq`) using the following call to Trimmomatic:

```
java -jar trimmomatic-0.32.jar PE in.1.fq in.2.fq \
    trimmo.1.fq out.up.1.fq trimmo.2.fq out.2.up.2.fq \
    ILLUMINACLIP:adapters.fasta:2:10:7:1 MINLEN:50
```

and the resulting paired reads `trimmo.1.fq` and `trimmo.2.fq` are retained. A file of Illumina adapters is distributed with IVA, but the user can provide their own file.

Next, perfect matches to PCR primers are removed using:

```
fastaq sequence_trim --revcomp trimmo.1.fq trimmo.2.fq \
    out.1.fq out.2.fq pcr_primers.fa
```

and the resulting paired reads `out.1.fq` and `out.2.fq` are then used for assembly. The user must provide a FASTA file of PCR primers.

2.3 Seed generation

When generating a seed kmer, the length is by default set to two-thirds of the read length, up to a maximum of 95. To save time, the first 100,000 reads are used as input to kmc, which is run with

```
kmc -fa -m4 -k $k -sf 1 -ci 200 -cs 1000000000 -cx 1000000000 \
    input_reads.fa kmc_out $PWD
kmc_dump kmc_out count
```

to make a file, `count`, of kmer counts, where

$$\texttt{\$k} = \min\left(\tfrac{2}{3} \times \text{read length}, 95\right).$$

2.4 Contig cleaning and merging

First, low quality contig ends are trimmed by removing bases that have more than 80% of their coverage on either the forward or reverse strand only. These were found by mapping the reads with SMALT with index options `-k 19 -s 11` and map options `-r 1 -x -y 0.9 -i 1000` and then running samtools mpileup with each of `--rf 0x10` and `--ff 0x10` to get the read depth on each strand.

Next, the set of contigs is aligned to itself using nucmer with settings

```
nucmer --maxmatch -p p contigs.fasta contigs.fasta
delta-filter -i 95 -l 100 p.delta > p.delta-filter
show-coords -dTlro p.delta.filter > out.coords
```

to make an output file of hits called `out.coords`. In particular, the minimum identity is 95% and minimum length is 100. In order of shortest to longest, any contig that has hits of total length at least 95% of its own length is discarded. Finally overlapping contigs are merged using nucmer hits linking one contig to another contig end. A merge is only made when there is exactly one hit between two contig ends, to minimize the introduction of new errors into the assembly.

3 Sample QC

3.1 Description of test data

172 Influenza virus and 68 HIV-1 clinical samples were initially considered (ENA accessions are in Tables S3 and S4). Prior to submission to the ENA, the reads were mapped to the human genome reference build GRCh37 with BWA version 0.5.10 (BWA-backtrack algorithm "aln") and option `-q 15`. Reads mapping to the human genome, and any relevant unmapped reads paired with a mapped read, were then removed with custom tool `AlignmentFilter` (https://github.com/wtsi-npg/illumina2bam).

3.2 Removing low quality samples

The analysis for this study started with the read sets from the ENA, which meant that host contamination was already removed from the reads. Many samples had large regions of the virus genome unrepresented in the reads due to failure of RT-PCR amplification. To determine which samples had unrepresented regions, the reads of each sample (after trimming Illumina adapter and PCR primer sequences as described in supplementary Section 2.2) were mapped to the closest reference using SMALT version 0.7.0.1 with index options `-k 13 -s 2` and map options `-r 1 -x -y 0.5 -i 1000`. In particular, this only required at least 50% of each read to map (`-y 0.5`). Any sample that did not have at least 90% of the reference genome positions covered by a minimum of 5 reads on each strand, and at least 1% of its reads mapped to the reference genome, was removed from further analysis. This was determined for each sample using the script

```
bam_is_genome_covered.pl in.bam 90 5 1
```

where `in.bam` contained the results of the SMALT mapping. After removing low quality samples, there were 98 Influenza virus and 42 HIV-1 samples remaining.

3.3 Reference databases

In order to assess the quality of each *de novo* assembly, Kraken was used to choose a closely related reference genome from public databases for each of the HIV-1 and Influenza virus samples. These reference genomes were solely used for evaluation purposes and were not used to aid the assembly process. The reference genomes were chosen using an automated method in order to be reproducible, and are sufficient for the analysis of the assemblies in this study. However, researchers may wish to manually build their own database to tailor to a particular project's needs.

3.3.1 HIV-1 reference database

HIV-1 reference genomes were chosen from the LANL database (http://www.hiv.lanl.gov/content/sequence/NEWALIGN/align.html) using the following method. The options Alignment type 'Compendium' and year '2012' were used and then 'Get Alignment' to download a multi-fasta alignment file called `HIV1_COM_2012_genome_DNA.fasta`. To make the file `hiv.ids`, the following command was run

```
hiv_get_good_genomes.pl HIV1_COM_2012_genome_DNA.fasta > hiv.ids
```

This selected only the genomes that had all 9 HIV genes (env, gag, nef, pol, rev, tat, vif, vpr, vpu) annotated and resulted in a file of 162 GenBank IDs. A Kraken database was made using the command

```
iva_qc_make_db --skip_viruses --add_to_ref hiv.ids HIV_db
```

Finally, the closest reference to each HIV-1 sample was chosen using Kraken by running the script

```
choose_ref.py HIV_db reads.fastq prefix_of_output_files.
```

A summary of the similarity between the assembly of each sample and the reference chosen is given in Tables S1 and S2.

3.3.2 Influenza reference database

Meta information for complete Influenza virus genomes was downloaded from the NCBI and 100 genomes of each of A and B type human Influenza virus were chosen using the script:

```
get_flu_genomes.pl > flu.ids
```

Similarly to that of HIV-1, a Kraken database was made with

```
iva_qc_make_db --skip_viruses --add_to_ref flu.ids Flu_db
```

and the closest reference to each Influenza virus sample was chosen using Kraken by running the script

```
choose_ref.py Flu_db reads.fastq prefix_of_output_files.
```

4 Benchmarking

Prior to assembly, all reads had Illumina adapter and PCR primer sequences trimmed using the methods described in supplementary Section 2.2. The default Illumina adapters file distributed with IVA was used with Trimmomatic. FASTA files of the PCR primer sequences are provided in the github repository (`hiv_pcr_primers.fa` and `flu_pcr_primers.fa`).

For each sample, the same trimmed reads were used as input to each of the assemblers IVA, PRICE, Trinity and VICUNA.

4.1 IVA

For each sample, IVA version 0.11.0 was run on paired FASTQ files `reads_1.fq` and `reads_2.fq` with:

```
iva --pcr_primers fasta_file_of_pcr_primers.fasta \
  --threads 8 -f reads_1.fq -r reads_2.fq Output_directory
```

Although the input reads already had PCR primers trimmed, the process is not perfect and using the option `--pcr_primers` makes IVA trim PCR primers off the ends of any contigs, as a final assembly stage.

Recall from the main text that IVA uses the longest available kmer of length k to extend a contig if that kmer appears at least 10 times and is at least four times as abundant as the next most common kmer of length k. In order to test the robustness of these values, we also ran IVA requiring that the kmer appeared at least five times and was at least two times as abundant as the next most common kmer. This required running IVA as above, but with the extra parameters `--ext_min_cov 5 --ext_min_ratio 2 --seed_ext_min_cov 5` and `--seed_ext_min_ratio 2`. The results in the main text refer to IVA run with the default settings, however, both sets of results are reported in this supplementary information. The default and alternative runs are referred to as 'IVA' and 'IVA.c5r2' throughout this text and supplementary tables.

4.2 VICUNA

VICUNA version 1.3 was run using a wrapper script:

```
vicuna-wrapper.pl -min_identify 80 \
  reads_1.fq reads_2.fq VICUNA 100 1000
```

where 100 and 1000 are the minimum and maximum insert sizes, and `min_identify 80` set the minimum percent identity to merge two contigs to 80%, instead of the default of 90%. These were the only values that were changed from the default settings. We also ran VICUNA with the default setting of `min_identify 90`, which on average produced lower quality assemblies. The numbers in the main text are with the value set to 80%, however, both sets of results are reported in the supplementary information. The two runs are referred to as 'VICUNA.80' and 'VICUNA.90' throughout this text and supplementary tables.

After assembling, we modified each contig name by retaining everything before the first whitespace with

```
awk '{print $1}' contig.fasta > contigs.fasta
```

This was to allow processing with downstream analysis tools.

4.3 PRICE

Version 1.2 of PRICE was used. PRICE needs starting seed sequences, which can be a subset of the input reads and using these makes the assembly truly *de novo*. We chose 200 reads at random (but evenly spaced throughout the file) from the FASTQ file `reads_1.fq` containing trimmed forward reads with:

```
fastq_to_equally_spaced_sample.py reads_1.fq seeds.out.fq
```

The seeds were converted to FASTA format (these FASTA files are available from the github repository). PRICE requires the insert size of the input reads. The median insert size was calculated from the SMALT output created when removing low quality samples (see the methods described in supplementary Section 3.2) using an in-house pipeline. The insert size used for each sample is given in Tables S3 and S4 (and supplementary files `table.S3.flu_samples.tsv` and `table.S4.hiv_samples.tsv`). PRICE was run with the following options:

```
PriceTI -a 8 -fpp reads_1.fq reads_2.fq $m 95  \
  -icf seed_reads.fasta 1 1 5 -nc 30 -dbmax 250 \
  -target 90 2 1 1 -o contigs.fasta
```

where `$m` was set to the appropriate median insert size for each sample.

We remark that we tried PRICE without the option `-target 90 2 1 1` and found it to exacerbate the problem of producing multiple copies of the same sequence over many contigs. This was as expected because the `-target` option, according to the manual, should limit the final contigs to extensions of the input seeds. Further, we tried PRICE without `-target 90 2 1 1` and also with 10 seed reads instead of 200. It exhibited the same behaviour of multiple contigs covering the same region of the genome (and since some of the 8 Influenza virus segments were not represented

in the 10 seed reads, those segments were understandably not present in the output contigs).

After assembling, only contigs of length at least 50bp were kept, and the contig names were modified in the same way as for VICUNA, using

```
fastaq filter --min_length 50 contigs.cycle30.fasta - \
    | awk '{print $1}' > contigs.fasta
```

As for VICUNA, this was to allow processing with downstream analysis tools.

The assembly of one Influenza sample (ERR732356) was manually terminated after running for six days. Since PRICE writes a FASTA file of contigs at the end of each iteration, we used the last file written as the final assembly. This was the 15th iteration.

4.4 Trinity

Trinity version 20140717 was run with the command:

```
Trinity --seqType fq --JM 16G \
  --left trimmo.pcr_trim_1.fq --right trimmo.pcr_trim_2.fq --CPU 8 \
  --output trinity.out
```

The final output from Trinity and the output from its first stage, 'Inchworm', were evaluated. To allow processing with downstream analysis tools the final contigs file output by Trinity was modified using

```
fastaq filter --min_length 50 Trinity.fasta - \
    | awk '{print $1}' > contigs.fasta
```

The Inchworm stage produces a file called `target.fa` and the above command was also applied to the file `target.fa` by substituting the input `Trinity.fasta` for `target.fa`.

5 Assembly validation

Two scripts for running assembly QC are distributed with IVA:

1. `iva_qc_make_db` – makes a custom database
2. `iva_qc` – runs all the QC analysis.

These both rely on Kraken being installed. We used version 0.10.4-beta of Kraken. The GAGE analysis code is also distributed with IVA, which we modified so that the minimum percent identity was parameterized and given a default of 80%, instead of the hard-coded 95%.

The QC script was run on each set of contigs with:

```
iva_qc -f trimmed_reads_1.fq -r trimmed_reads_2.fq \
  --embl_dir EMBL contigs.fasta output_prefix
```

where trimmed_reads_1.fq and trimmed_reads_2.fq were the forward and reverse trimmed reads and EMBL was the directory of EMBL files chosen to be the closest reference (as described earlier).

The complete results of running the QC are given in Tables S5 and S6 (and supplementary files table.S5.qc_summary.flu.tsv and table.S6.qc_summary.hiv.tsv). A summary of the results of all assemblies is given in Tables S1 and S2. Box plots of the proportion of genome assembled and success of annotation transfer are given in Figure S3.

6 Run time and memory usage

None of the assemblers had significant memory requirements. The values used for peak memory usage were those reported by the compute farm job scheduling software Platform Load Sharing Facility (LSF). It polls the memory usage every minute and reports the maximum value when a job finishes.

The option --JM 16G was used with Trinity, which requested 16GB of Java memory when running Jellyfish (a k-mer counting program used as part of the Trinity pipeline). Although for some samples a lower value could have been used to reduce the peak memory usage, we found that many of the assemblies crashed with --JM 8GB and therefore --JM16 was used for all samples. We do not report values for the Inchworm stage of Trinity because it is one part of the entire Trinity assembly pipeline and therefore the numbers were not available.

The run time varied between assemblers and samples. See Figure S4 for summary plots of the CPU and memory usage. The complete data are in Tables S7 and S8 (and supplementary files table.S7.resources_flu.tsv and table.S8.resources_hiv.tsv). Note that IVA, PRICE and Trinity were used with 8 threads and VICUNA has no threading option.

7 Example assemblies

Typical results of assembling an Influenza virus sample are shown in supplementary Figures S5 and S6. These plots are produced by the script iva_qc. IVA assembles all segments, with most segments assembled into one unique contig. PRICE and VICUNA assemble most segments, but with many duplications. Inchworm also has many duplications, but most of these are removed in the final output of Trinity. Each plot shows the following information.

- Vertical grey lines mark the boundaries between segments of the genome (only applicable to Influenza virus).
- The top panel shows the contigs aligned to the closest reference genome (chosen for each sample as described earlier), using nucmer hits. There is one row per contig, and one column per genome segment. More than one rectangle on a row represents a chimeric contig. Blue means that each part of the contig had a single nucmer match to the reference. Otherwise, the contig is coloured red. Light and dark corresponds to forward and reverse orientation respectively.

- The middle panel is in two sections, the upper section shows contig information and the lower section shows read information. See Figure S7 for an example where all of the tracks are visible (they are not all visible in Figure S5). The upper three tracks show contig coverage of the reference. The first and second tracks (black) show presence and absence of contig coverage. The third track (red) shows absence of contig coverage, but where there was at least 5X read coverage on each strand, i.e. assembly should have been possible. The tracks are not all visible in Figure S5 because there was good read coverage across the entire genome.

- The lower three tracks of the middle panel show properties of the read depth. The first track (black) shows where there was at least 5X read depth on both strands. The second and third tracks (red) show read depth less than 5X on the forward and reverse strands respectively.

- The bottom panel shows line plots of the read depth on the forward and reverse strand above and below the y axis respectively.

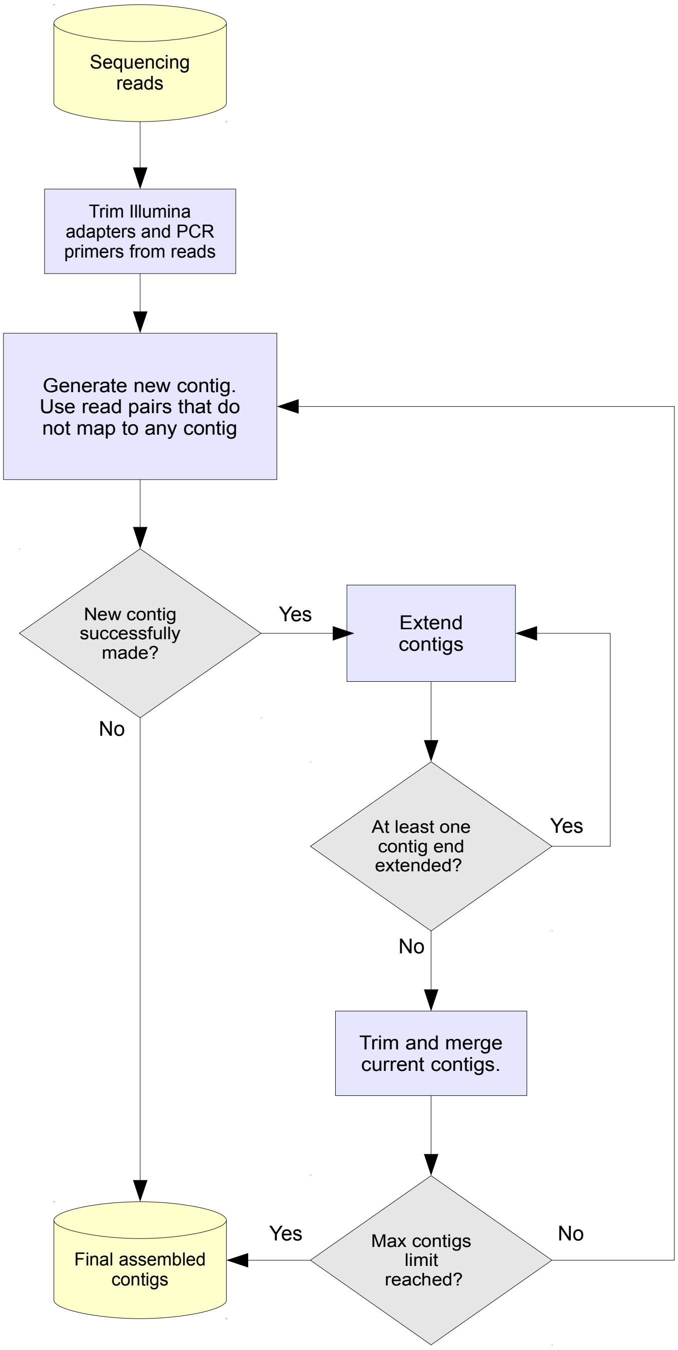

Figure S1: Flowchart of the IVA assembly algorithm

	IVA	IVA.c5r2	PRICE	Inchworm	Trinity	VICUNA.80	VICUNA.90
Ideal assemblies (%)[1]	57.1	57.1	11.9	0.0	14.3	2.4	0.0
Mean reference bases assembled (%)	97.9 (2.53)	98.1 (2.51)	97.2 (4.98)	98.4 (2.39)	89.8 (20.9)	98.3 (2.37)	98.4 (2.39)
Longest contig(s) sum (% of reference)	99.9 (7.64)	101.5 (3.81)	94.4 (25.96)	90.1 (20.4)	76.2 (29.98)	99.5 (11.03)	101.5 (7.0)
Assembly length (% of reference)	110.5 (22.99)	109.0 (20.05)	127.2 (33.36)	312.9 (97.19)	154.0 (79.7)	146.1 (38.09)	160.7 (54.16)
Mean duplication rate[2]	0.1 (0.14)	0.1 (0.13)	0.2 (0.23)	1.8 (0.93)	0.5 (0.6)	0.2 (0.22)	0.3 (0.3)
Mean % annotation transferred	99.0 (5.04)	98.0 (8.04)	90.0 (17.91)	98.7 (4.11)	86.2 (22.62)	97.3 (9.24)	99.2 (3.55)
Total assembly errors[3]	1	4	4	2	0	1	3
Mean per-sample identity to reference (%)[4]	91.0 (1.33)	91.0 (1.44)	91.1 (1.41)	91.1 (1.4)	91.2 (1.41)	91.2 (1.38)	91.1 (1.34)

[1] the entire genome must be assembled into a unique contig.
[2] Number of duplicated reference bases reported by GAGE divided by the length of the reference.
[3] An error is an inversion, relocation or translocation reported by GAGE. Numbers reported are the total across all assemblies.
[4] as reported by GAGE.

Table S1: Summary of HIV-1 QC results. Numbers in parentheses are the standard deviations. See Figures 2a, 2b, S2 (an expanded version of Figure 2) and S3 for Box plots.

	IVA	IVA.c5r2	PRICE	Inchworm	Trinity	VICUNA.80	VICUNA.90
Ideal assemblies (%)[1]	21.4	18.4	0.0	0.0	1.0	0.0	0.0
Mean reference bases assembled (%)	98.8 (2.84)	99.0 (2.66)	89.8 (13.81)	99.6 (1.39)	97.6 (4.64)	94.3 (4.38)	92.4 (4.81)
Longest contig(s) sum (% of reference)	96.6 (6.51)	99.0 (5.49)	91.8 (16.61)	94.9 (10.18)	94.4 (8.97)	82.0 (10.95)	75.7 (11.16)
Assembly length (% of reference)	107.2 (8.09)	107.7 (8.26)	290.7 (92.12)	229.4 (48.32)	131.7 (25.92)	136.9 (21.0)	135.4 (18.8)
Mean duplication rate[2]	0.1 (0.07)	0.1 (0.07)	1.8 (0.84)	0.4 (0.21)	0.3 (0.27)	0.4 (0.2)	0.4 (0.18)
Mean % annotation transferred	99.0 (2.87)	99.1 (2.63)	92.1 (11.12)	98.3 (3.14)	96.1 (4.45)	95.3 (5.77)	92.9 (7.84)
Total assembly errors[3]	0	6	6	4	0	0	0
Mean per-sample identity to reference (%)[4]	99.0 (1.07)	99.0 (1.07)	98.8 (1.1)	99.0 (1.0)	99.0 (1.03)	98.9 (1.1)	99.0 (1.06)

[1] each segment must be assembled into a unique contig.
[2] Number of duplicated reference bases reported by GAGE divided by the length of the reference.
[3] An error is an inversion, relocation or translocation reported by GAGE. Numbers reported are the total across all assemblies.
[4] as reported by GAGE.

Table S2: Summary of Influenza QC results. Numbers in parentheses are the standard deviations. See Figures 2a, 2b, S2 (an expanded version of Figure 2) and S3 for Box plots.

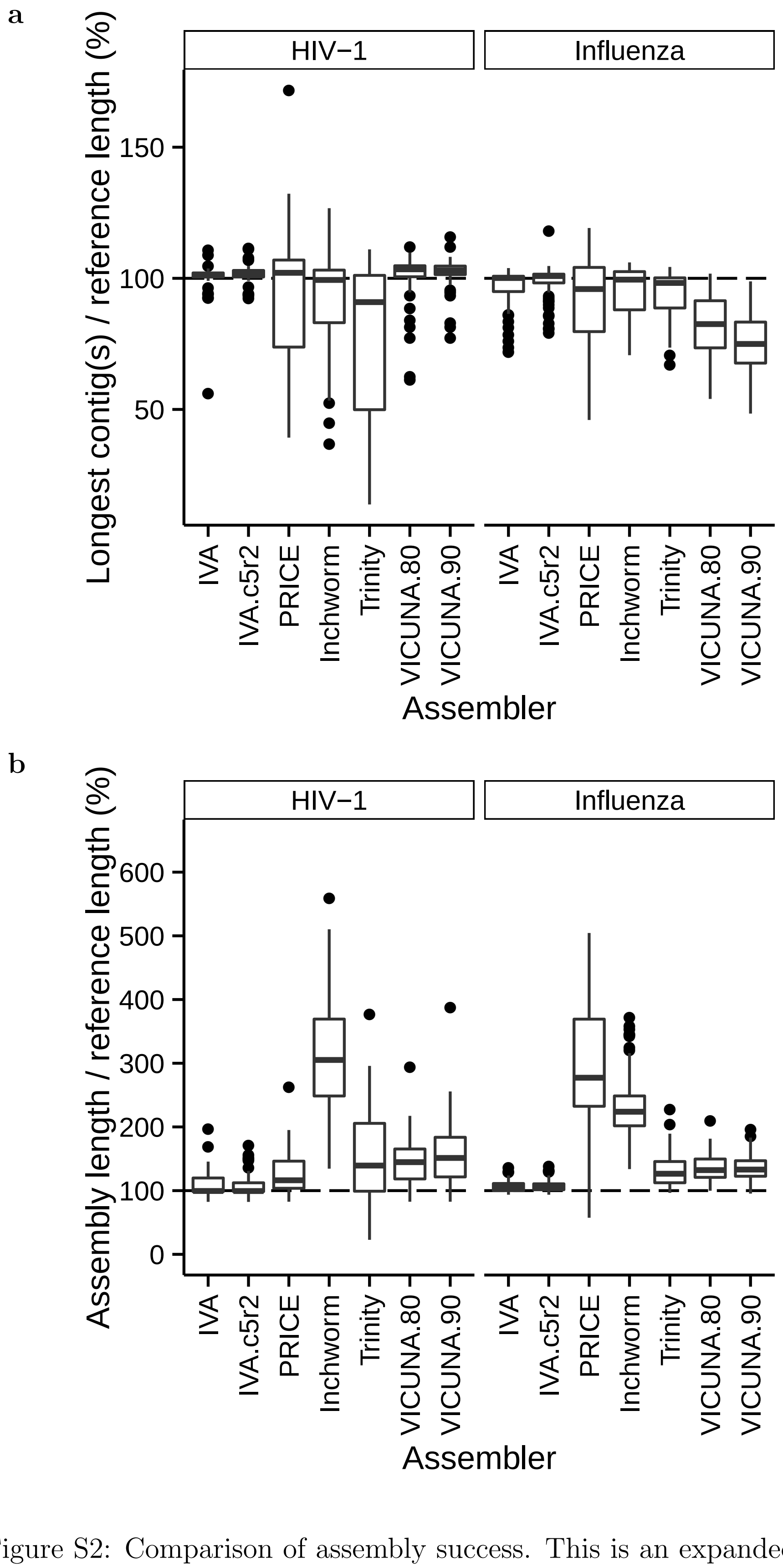

Figure S2: Comparison of assembly success. This is an expanded version of Figure 2. (a) For each segment of the reference, the longest matching contig was found. This plot shows the total length of these contigs for each assembly, as a percentage of the reference length. (b) Total assembly lengths, excluding contamination by only counting contigs that match the reference, as a percentage of the reference length.

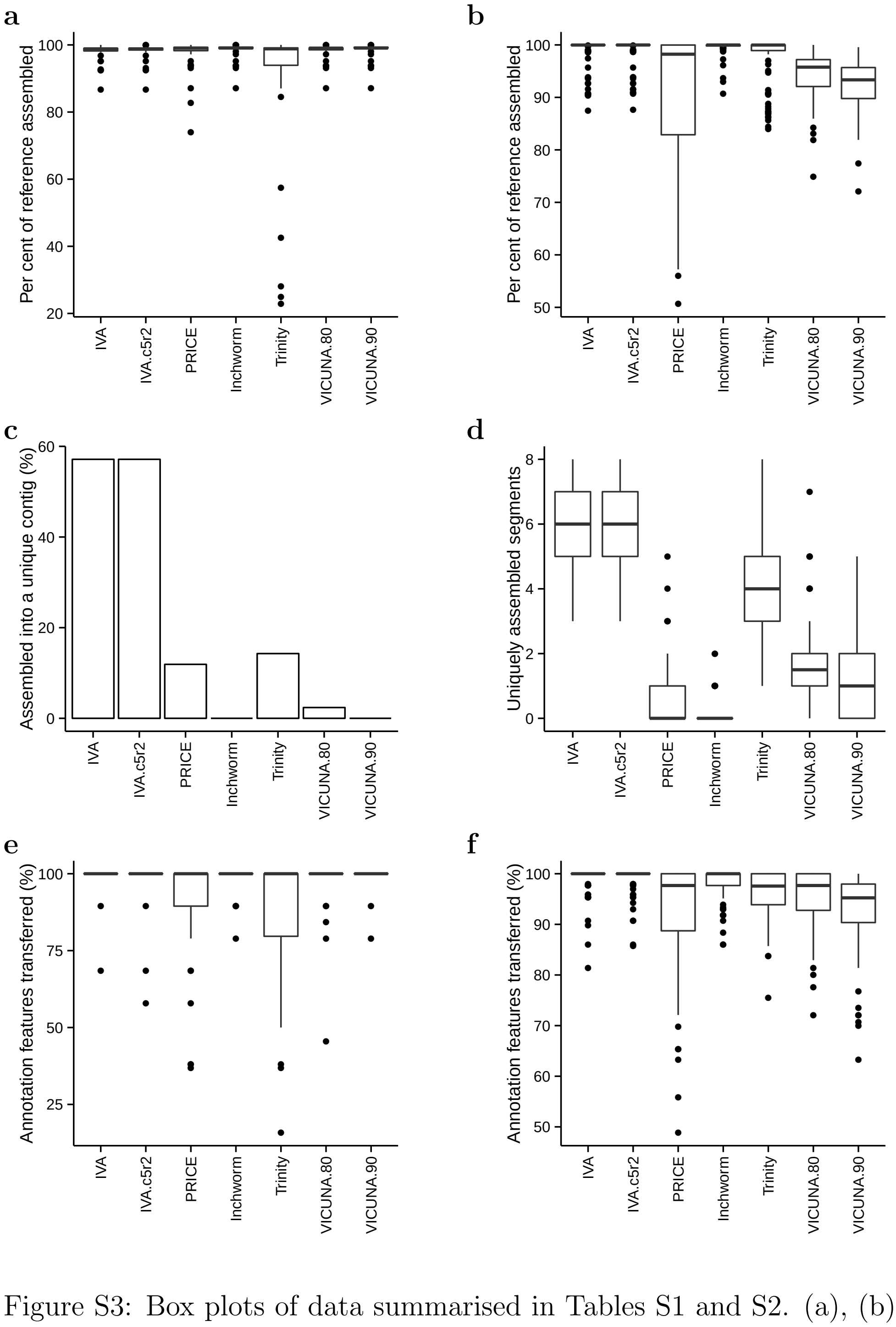

Figure S3: Box plots of data summarised in Tables S1 and S2. (a), (b) Per cent of genome assembled, using GAGE output, for (a) HIV-1 and (b) Influenza. (c) Per cent of HIV-1 samples that were assembled into a single unique contig. (d) Number of segments assembled into a single unique contig, for each Influenza sample. (e), (f) Per cent of annotation elements transferred from reference for (e) HIV-1 and (f) Influenza.

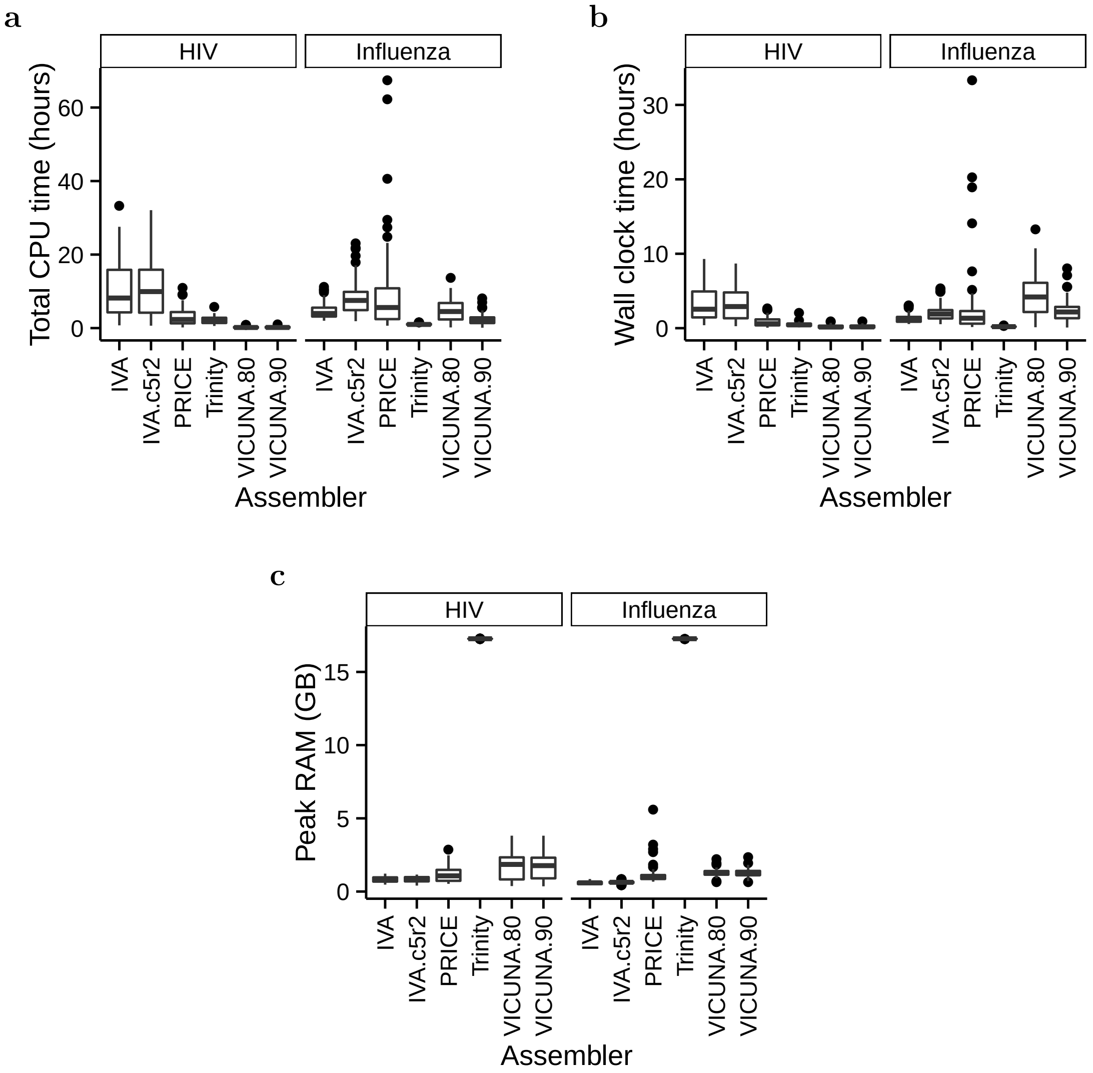

Figure S4: Resource usage of the assemblers per sample. (a) Total CPU time. Two PRICE outliers were removed from the Influenza plot, with values 177 and 650. (b) Wall clock time. One PRICE outlier was removed from the Influenza plot, with value 144. (c) Peak RAM usage.

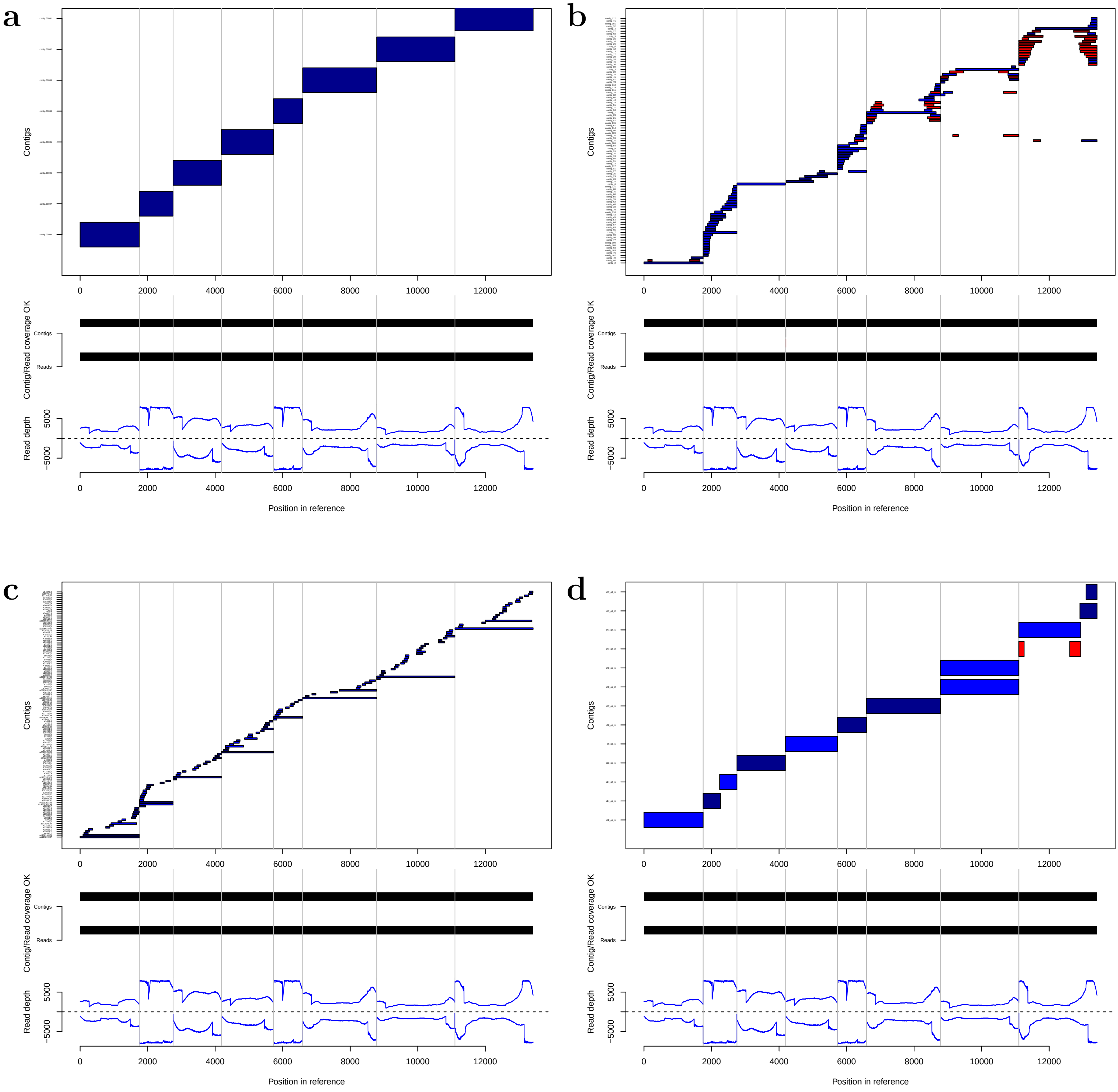

Figure S5: Example Influenza virus assemblies for sample ERR732276. (a) IVA, (b) PRICE, (c) Inchworm, (d) Trinity. The corresponding plot for VICUNA is in Figure S6. See text for an explanation.

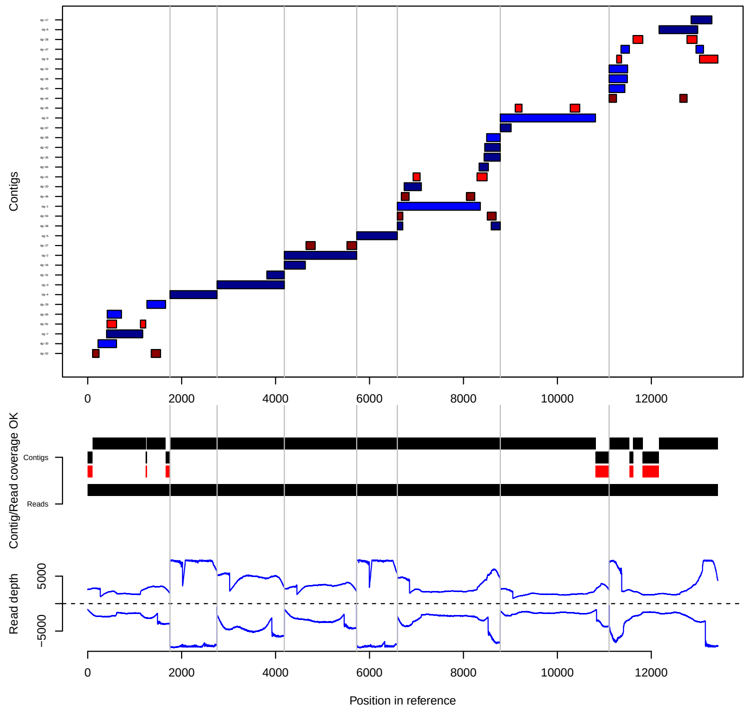

Figure S6: VICUNA assembly for Influenza virus assembly sample ERR732276. See Figure S5 for plots with the other assemblers.

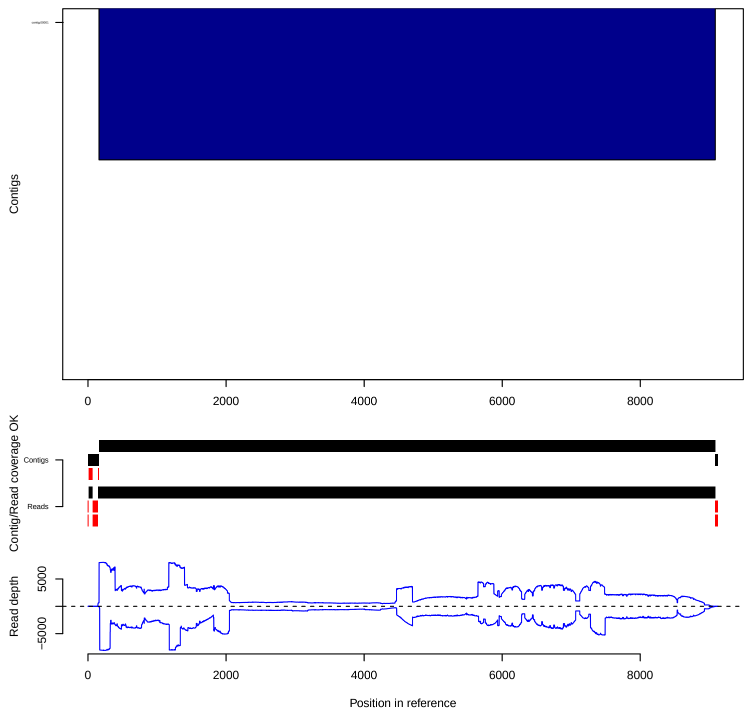

Figure S7: IVA contig layout, contig coverage and read depth for HIV sample ERR732130. See main text for an explanation.
